# Proangiogenic effects of peritumoral adipose tissue in kidney cancer

**DOI:** 10.3389/fmed.2025.1652589

**Published:** 2025-09-09

**Authors:** Matías Ferrando, Nadia Bannoud, Fiorella Campo-Verde-Arbocco, Leonardo Rafael Romeo, Constanza Matilde López-Fontana, Rubén Walter Carón, Flavia Alejandra Bruna, Diego Omar Croci, Virginia Pistone-Creydt

**Affiliations:** ^1^Institute of Medicine and Experimental Biology of Cuyo (IMBECU), Mendoza Scientific and Technological Center, National Council for Scientific and Technical Research (CONICET), National University of Cuyo, Mendoza, Argentina; ^2^Laboratory of Glycobiology and Vascular Biology, Scientific and Technological Center of Mendoza, Institute of Histology and Embryology of Mendoza (IHEM), National Council for Scientific and Technical Research (CONICET), Mendoza, Argentina; ^3^Faculty of Medical Sciences, Institute of Physiology, National University of Cuyo, Centro Universitario, Mendoza, Argentina; ^4^Urocuyo, Mendoza, Argentina; ^5^Institute of Technology of Buenos Aires (ITBA), Buenos Aires, Argentina

**Keywords:** human adipose tissue, renal cancer, angiogenesis, epithelial-stromal interactions, endothelial cells migration

## Abstract

**Background:**

Tumor growth and metastasis require the interaction of tumor cells with the stromal environment. Angiogenesis is a necessary process for tumor growth and metastasis. Previously we showed that the conditioned media (CMs) of human renal adipose tissue from patients with renal tumors (hRAT) increases the migration of tumor and non-tumor renal epithelial cells compared to CMs of normal adipose tissue (hRAN).

**Methods:**

We evaluated: (1) mRNA expression of hypoxia inducible factor (HIF) 1α, HIF2α, and vascular endothelial growth factor (VEGF) in hRAN and hRAT, by qRT-PCR; (2) protein expression VEGF in hRAN-CMs and hRAT-CMs, by ELISA; (3) migration of endothelial cells (ECs) incubated with hRAN-CMs and hRAT-CMs, by wound healing assay and transwells; and (4) tube formation by ECs, incubated with hRAN- and hRAT-CMs.

**Results:**

We found a higher expression of HIF1α, HIF2α in hRAT vs. hRAN explants (*p* < 0.05). Also, we observed a close to significance trend toward higher VEGF protein expression (*p* = 0.052) in hRAT-CMs vs. hRAN-CMs explants. In addition, we found that hRAT-CMs significantly induced the migration of ECs compared to hRAN-CMs (*p* < 0.05). Finally, an increased tubulogenesis of ECs incubated with hRAT-CMs vs. hRAN-CMs was observed (*p* < 0.05).

**Conclusion:**

We show that renal peritumoral adipose tissue secretes VEGF and promotes angiogenesis on HUVEC cell lines, suggesting that VEGF, among other factors, may contribute to this effect. This proangiogenic stimulus would promote the vascularization of the tumor, favoring its growth and metastasis.

## 1 Introduction

Renal cell carcinoma (RCC) is one of the most common genitourinary cancers (1). Clear cell renal cell carcinoma (ccRCC) represents the most prevalent and aggressive form of RCC, accounting for 80%–90% of kidney tumors. It is highly resistant to chemotherapy and prone to metastasis, with approximately 30% of cases diagnosed at advanced or metastatic stages (ccRCC) and an additional 20%–30% developing metastasis later in the course of the disease (2).

Angiogenesis, known as *de novo* vessel formation, is a fundamental process (along with neovasculogenesis) for both development and normal performance of every tissue. During the “avascular” phase, tumors obtain nutrients and oxygen through adjacent vessels. Thus, to ensure active proliferation and growth without nutritional restrictions the activation of angiogenic processes is mandatory (3, 4).

The tumor’s vicinity is composed of blood and lymphatic vessels, fibroblasts, stem cells and adipocytes (5). Adipose tissue (AT) plays a central role in storing and releasing energy to satisfy nutritional requirements. Furthermore, it is now widely recognized as an organ with endocrine and immunomodulatory functions, contributing to human physiology through both localized and systemic mechanisms (6). Recent investigations have revealed that growth factors and cytokines secreted by AT, significantly influence the progression of various diseases, including cancer (7–15). Proteins secreted by peritumoral adipose tissue engage in paracrine interactions with tumor cells. Moreover, the proximity of AT to cancer cells has been shown to alter the structure and function of peritumoral adipose tissue (11–16). The influence of tumor microenvironment is reflected in both mature adipocytes and adipose stem cells (17). Tumor cells stimulate the dedifferentiation of adipocytes, and in this scenario, adipocytes rearrange their cytoskeleton and develop a fibroblast-like phenotype (18). Peritumoral adipocytes, in contrast to normal adipocytes, exhibit an altered phenotype and are called cancer-associated adipocytes (CAA). Peritumoral mammary adipocytes at the invasive tumor front have been shown to be smaller, undergo delipidation, and turn brown (19–21).

The reduction of oxygen levels in the environment (hypoxia) is one of the main triggers of angiogenesis and regulates the activation of a family of Hypoxia-inducible transcription factors (HIFs). HIFs are pivotal in hypoxic tumor cells, driving inflammation and angiogenesis (22). These proteins consist of three α-subunits (HIF1α, HIF2α, and HIF3α) and one β-subunit (HIF1β), which serves as a heterodimerization partner of the HIFα subunits. Under normoxic conditions the gene product of Von Hippel Lindau (VHL), pVHL, forms the molecular complex E3 ubiquitin ligase and targets HIFs for proteosomal degradation. In absence of VHL, hypoxia-responsive genes are upregulated due to HIFs. HIF1α and HIF2α are expressed in different tissues and regulate target genes involved in angiogenesis (like VEGF), cell proliferation and inflammation, epithelial-to-mesenchymal transition, apoptosis, metastasis, and tumor invasion, and their expression is associated with different disease states (23–25).

In patients with mccRCC, perirenal adipose tissue exhibits an increased presence of M2 macrophages, which secrete angiogenic factors and cytokines, thereby promoting angiogenesis and cancer progression (26). Nevertheless, it remains unclear whether renal peritumoral adipose tissue directly regulates local angiogenesis through the release of factors into the tumor microenvironment.

Our research group recently showed that conditioned media (CMs) derived from human renal adipose tissue in renal tumor patients (hRAT) enhances the migration of both tumor and non-tumor renal epithelial cells when compared to CMs from normal tissue (hRAN) (14). The proteolitic degradation of the extracellular matrix (ECM) is a adipose key step in the angiogenic process (4). We demonstrated that adipose tissue, adjacent to renal tumors; secrete different factors like versican, among others, that regulate cell-ECM adhesion (12). In addition, we showed that CMs derived from human renal adipose tissue in renal tumor (hRAT) enhances the migration of epithelial cell lines (14, 15).

Given the above mentioned results, in the present work we hypothesize that peritumoral adipose tissue secretes soluble factors into the tumor’s environment with pro-angiogenic capacity, which could ultimately promote tumor development and metastasis. In the present study, we analyzed the differential expression of HIF1α, HIF2α, and VEGF in hRAN and hRAT. Furthermore, we compared the effects of hRAT-CMs and hRAN-CMs on promoting cell migration and tube formation in primary cultures of human umbilical vein endothelial cells (HUVECs).

## 2 Methods

### 2.1 Reagents

Reagents were from Sigma Chemical Co (St. Louis, MO, USA), tissue culture flasks, dishes, and multi-well plates were from Falcon Orange Scientific (Graignette Business Park, Belgium), culture media for cell lines was from Gibco BRL (Carlsbad, CA, USA), culture media for tissues, VEGF and bovine fetal serum were from Sigma Aldrich (St. Luis, MO, USA).

### 2.2 Sample collection and handling

Patients with suspected kidney cancer or healthy kidney donors were enrolled. After signing the informed consent, subjects were interviewed using a standard questionnaire that requested information about socio-demographic, medical, and lifestyle factors. Human adipose tissue explants from cancerous kidneys were obtained from patients to whom a partial or total nephrectomy was performed, who had not received previous chemotherapy or radiotherapy treatment. Human adipose tissue explants from normal kidneys were obtained from live kidney donors. Perirenal adipose tissue biopsies were taken as follows: (1) in the case of living kidney donors, the adipose tissue fragment was taken 1 cm away from the kidney from the middle zone (middle pole); (2) in the case of patients with kidney cancer, the fragment of adipose tissue was taken 1 cm from the kidney, also considering the location of the tumor. Trying, in all cases, to take the sample 1 cm from the location of the tumor, getting as close as possible to the middle zone. In all cases, biopsies were taken distant from the adrenal gland (sources of norepinephrine).

The median body mass index (BMI) of patients was: 26.8 kg/m^2^ for patients with renal tumor (hRAT), and 24.9 kg/m^2^ for living kidney donors (hRAN). BMI (kg/m^2^) was calculated as weight (kg) divided by height (m) squared. Of the living kidney donors, 57% are female and 43% are male, with a mean age of 44 years (+/− 7). Of the ccRCC patients, 27% are female and 73% are male, with a mean age of 56 years (+/− 14). All patients are from the population of the Province of Mendoza, Argentina.

Samples were transported in PBS and processed immediately; a fragment of each one was stored at −80 °C, another fragment was processed under a sterile laminar flow hood. The project was approved by the Medical School’s ethics committee (Universidad Nacional de Cuyo, Argentina, EXP-CUY: 11722–2016) according to the Declaration of Helsinki of experimentation with human subjects. All patients gave their informed consent to undergo tissue harvesting for this research (14, 15).

### 2.3 Gene expression by RT-qPCR analysis

Total RNA was extracted from 100 mg of tissue (hRAN *n* = 8; hRAT *n* = 8) using Trizol reagent (Invitrogen, Carlsbad, CA, USA) and quantified according to its absorbance at 260/280 nm. Two micrograms of total RNA were reverse transcribed at 37 °C using random hexamer primers and Moloney murine leukemia virus retrotranscriptase in a 20 μL reaction mixture. The RNA was first denatured at 70 °C for 5 min. in the presence of 2.5 μg of random hexamer primers (Thermo Fisher Scientific, Carlsbad, CA, USA). For the subsequent RT reaction, the following mixture was added: RT buffer [50 mM Tris–HCl (pH8.4), 75 mM KCl, 3 mM MgCl2], 0.5 mM dNTPs, 5 mM DTT, 200 units M-MLV Reverse Transcriptase (Thermo Fisher Scientific, Carlsbad, CA, USA). The reaction was incubated at 37 °C for 50 min., next, the reaction was inactivated by heating at 70 °C for 15 min. The cDNA was stored at −20 °C. The mRNA content of each gene was estimated by RT Real-Time PCR using specific primers described in Table 1. The PCR reactions were performed using a Corbett Rotor Gene 6000 Real-Time Thermocycler (Corbett Research Pty Ltd. Sydney, Australia) and the HOT FIREPol® DNA Polimerase (Solis BioDine, Tartú, Estonia). The PCR reactions were initiated with 15 min incubation at 95 °C, followed by 40 cycles of 95 °C for 30 s, 30 s at the annealing temperatures shown in Table 1 and 72 °C for 30 s. Melt curve analysis was used to check that a single specific amplified product was generated. Real time quantification was monitored by measuring the increase in fluorescence at the end of each amplification cycle, according to the manufacturer’s protocol. Relative expression was determined using the Comparative Quantitation method of normalized samples in relation to the expression of a calibrator sample (control sample). Each PCR run included a no-template control and a sample without reverse transcriptase. All measurements were performed in duplicate by sample of two independent experiments. The reaction conditions and quantities of cDNA added were calibrated so that the assay response was linear with respect to the amount of input cDNA for each pair of primers. Relative levels of mRNA were normalized to the GAPDH reference gene (14, 15).

**Table 1:** Primer pair sequences are shown for the forward (F) and reverse (R) primers used to measure mRNA abundance by RT-qPCR.

Gen	Forward (5′–3′)	Reverse (5′–3′)	Annealing T°	Amplicon size (pb)	Gene Bank
HIF-1α	TTCACCTGAGCCTAATAGTCC	CAAGTCTAAATCTGTGTCCTG	55 °C	151	NM_001530.4
HIF-2α	TTGCTCTGAAAACGAGTCCGA	GGTCACCACGGCAATGAAAC	55 °C	91	NM_001430.5
VEGF	AGGGCAGAATCATCACGAAGT	GGTCACCACGGCAATGAAAC	55 °C	75	NM_001287044.2
GADPH	GGAGCGAGATCCCTCCAAAAT	GGCTGTTGTCATACTTCTCATGG	55 °C	197	NM_002046.7

### 2.4 Preparation of conditioned media (CMs) from hRAN and hRAT

Adipose tissues were washed with cold PBS 1× (Gibco, USA) and weighed. hRAN or hRAT were plated in culture flasks with M199 culture medium (Invitrogen™; 1 g tissue/10 mL M199) and incubated for 1 h at 37 °C in 5% CO_2_. After that, the medium was removed and replaced with fresh medium and the tissues were incubated for 24 h. Afterwards, the supernatant was collected and filtered with sterile 0.22 μm membranes. Then, supernatants were aliquoted into 1 mL fractions and immediately stored at –80 °C. The control-CMs were obtained from the collection of the serum-free M199 medium after 24 h of incubation in a culture flask at 37 °C in 5% CO_2_ (14, 15).

### 2.5 VEGF protein quantification

Levels of VEGF in hRAN- (*n* = 10) or hRAT-CMs (*n* = 19) were determined using enzyme-linked immunoassay (ELISA) kit (R&D Systems, Minneapolis, MN, USA). In brief, high-binding 96-well microplates (Costar; Corning) were coated with capture Ab (2 μg/mL purified rabbit anti-VEGF polyclonal IgG) in 0.1 M sodium carbonate, pH 9.5. After incubation for 18 h at 4 °C, wells were rinsed three times with wash buffer (0.05% Tween-20 in PBS) and incubated for 1 h at room temperature with blocking solution [2% Bovine Serum Albumin (BSA) in Phosphate buffer saline (PBS)] Samples and standards (100 μL) were diluted in 1% BSA–Tween-20 and incubated for 18 h at 4 °C. Plates were then washed and incubated with 100 ng/mL biotinylated detection Ab (purified rabbit anti-VEGF polyclonal IgG) for 1 h. Plates were rinsed three times before adding 0.33 μg/mL HRP-labeled streptavidin (Sigma-Aldrich) for 30 min. After washing, 100 μL 3,3′,5,5′-Tetramethylbenzidine (TMB) solution (0.1 mg/mL tetramethylbenzidine and 0.06% H_2_O_2_ in citrate–phosphate buffer, pH 5.0) was added to plates. The reaction was stopped by adding 2 N H_2_SO_4_. Optical densities were determined at 450 nm in a Multiskan MS Microplate Reader (Thermo Fisher Scientific). A standard curve ranging from 2.5 to 160 ng/mL VEGF was run in parallel (27).

### 2.6 Isolation of HUVECs

Isolation of HUVECs from umbilical cords was performed essentially as described in Bannoud et al. (27), following approval by the Ethics Committee from the Faculty of Medicine, National University of Cuyo (Mendoza, Argentina) (ref. EXP-CUY: 4693–2018 and updated in 2019); and by the Research Ethics Committee from the Central Hospital of Mendoza (Mendoza, Argentina) (Approval record: 10/2022).

### 2.7 Wound healing assay

The 96-well plates were coated with Geltrex® and maintained at 37 °C for 30 min. After that, excess gelatin was recovered, and cells were seeded (2.5×10^4^ cells per well). HUVEC cells were grown on complete medium RPMI 15% FBS with 10 ng/mL VEGF. Confluent cell monolayers were wounded with a pipette tip, washed twice with PBS and treated with hRAN-CM (*n* = 6), hRAT-CM (*n* = 6), or M199 growth medium as negative control, all diluted with RPMI 2% FBS and positive control (RPMI 15% BFS with 20 ng/mL VEGF). Images at time zero (0 h) were captured to record the initial width of the wounds. The recovery of the wounded monolayers due to cell migration toward the denuded area was evaluated after 6 and 12 h. The images were acquired by an inverted phase-contrast microscope (Olympus CKX-41) using a 4× objective. Quantification was performed using ImageJ analysis software (NIH, Bethesda, MD, USA) by a polygon selection mode and determining the percentage of the wounded area at 6 or 12 h respect to control (0 h) (14, 15).

### 2.8 Transwell migration assay

HUVEC cells (3×10^4^ cells/0.2 mL) were placed into the top transwell with 8 μm pore membranes (COSTAR® cat. #03123015). They were then incubated with hRAN-CM (*n* = 6), hRAT-CM (*n* = 6), negative control (M199 growth medium *n* = 6), all diluted with RPMI 2% FBS, and positive control (RPMI 15% FBS with 20 ng/mL VEGF *n* = 3) and allowed to transmigrate across the porous membrane for 18 h. At the end of the assay, inserts were removed, and the cells were stained with a 0.1% crystal-violet in 20% methanol (Sintorgan®) solution for 10 min. The inserts were washed in distilled water and the cells on the upper membrane surface were removed with a cotton swab. The air-dried membranes were viewed under 20× magnification and migrated cells were counted in five randomly chosen fields per membrane (14, 27).

### 2.9 Tubulogenesis assay

The formation of capillary-like tubular structures was assessed in Geltrex-coated plates as described (26). In brief, HUVECs (1.5 × 10^4^ cells) were mixed with different CMs, and then plated on Geltrex-coated 96-well plates at 37 °C for 12 h or, alternatively, on Geltrex-coated *μ*-slide angiogenesis (Ibidi®) for 6–8 h. Capillary-like tubular structures were visualized by phase-contrast microscopy and recorded by counting the number of tubules (closed areas) per well in a phase-contrast microscope (Nikon E-100) (27).

### 2.10 Statistical analysis

GraphPad Prism version 8 (GraphPad Software Inc.) was used for statistical analysis. For comparisons between two datasets we performed *t*-test with Mann–Whitney or Welch’s corrections as appropriate. For multiple comparisons, one-way ANOVA followed by Tukey’s or Dunnett’s posttest (parametric analysis) or the Kruskal–Wallis test followed by Dunn’s posttest (nonparametric analysis), were used. *p*-values < of 0.05 or less were considered significant.

## 3 Results

### 3.1 hRAT showed an increase in gene expression of HIF-1α and HIF-2α compared to hRAN

**Figure 1:**
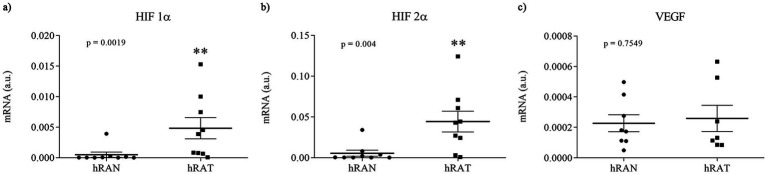
Relative fold expression of HIF 1α, HIF 2α, and VEGF, gene expression from hRAN and hRAT. The mRNA profiles of HIF 1α **(a)**, HIF 2α **(b)** and VEGF **(c)**, from different adipose tissues were analyzed by qRT PCR and normalized by their relative ratio to GAPDH. Horizontal bars represent the geometric mean of each data set and vertical bars indicate SEM. *p < 0.05, **p < 0.01. The *n* corresponds to eight patient samples from each group, and the experiments were performed in triplicate.

We evaluated gene expression profiles of angiogenesis regulatory factors in different fragments of adipose tissue. To do so, we measured mRNA levels of HIF-1α, HIF-2α, and VEGF in AT from normal and tumor kidney samples. We found higher gene expression of HIF1α and HIF2α in hRAT vs. hRAN explants (Figures 1a,b, *p* < 0.01). No statistically significant differences were found in VEGF mRNA expression among groups (Figure 1c).

### 3.2 The expression of VEGF was higher in hRAT-CMs than hRAN-CMs

**Figure 2:**
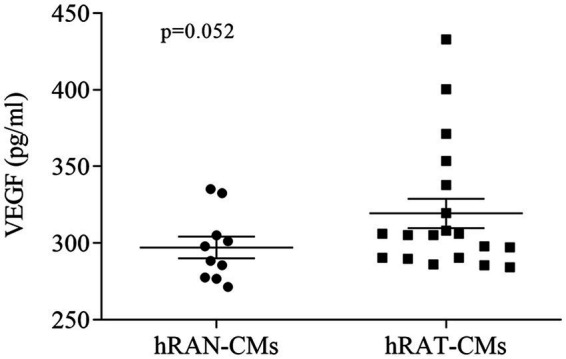
VEGF protein secretion from hRAN and hRAT CMs. VEGF secretion was determined by ELISA (R&D systems) in hRAN (*n* = 10) and hRAT (*n* = 19) conditioned media. Horizontal bars represent the geometric mean of each data set and vertical bars indicate SEM. *p* = 0.052. The *n* corresponds to eight patients in each group, and the experiments were performed in triplicate.

In order to evaluate the secretion of VEGF protein in hRAN- and hRAT-CMs, we conducted an ELISA assay. We observed a close to significance trend toward higher VEGF secretion in hRAT-CMs compared to hRAN-CMs (Figure 2, *p* = 0.052).

### 3.3 hRAT-CMs promoted migration of ECs

**Figure 3:**
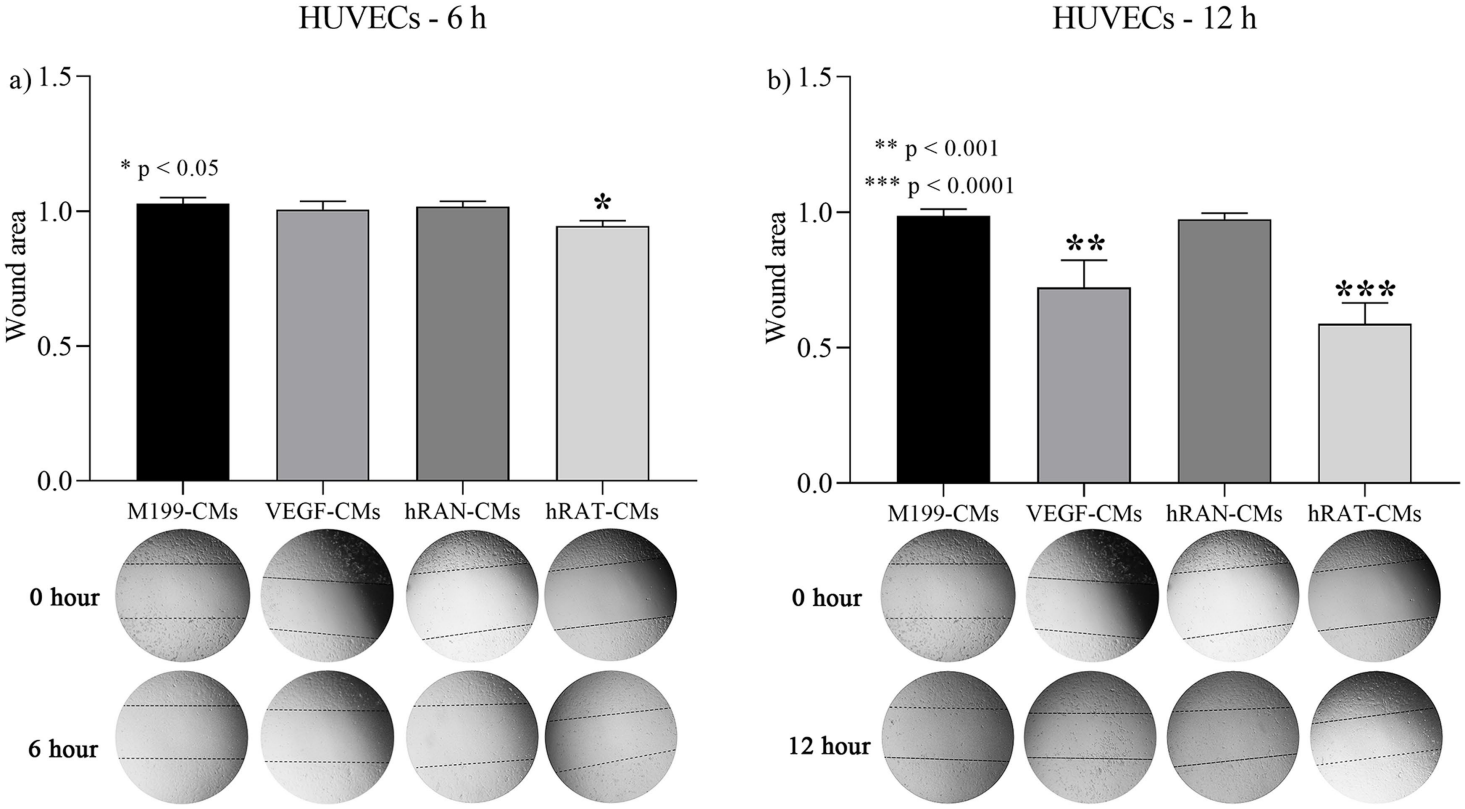
Effect of CMs from hRAN and hRAT on migration of HUVEC cells. The histogram shows the ratio of 6 h/ 0 h **(a)** or 12 h/ 0 h **(b)** cutting area. Horizontal bars represent the geometric mean of each data set and vertical bars indicate SEM. *p < 0.05 hRAT CMs vs. control M199-, control VEGF-, hRAN-CMs; **p < 0.001 control VEGF-CMs vs. control M199-, hRAN-CMs; ***p < 0.0001 hRAT-CMs vs. control M 199, hRAN-CMs. The *n* corresponds to six patient samples from each group, and the experiments were performed in triplicate.

To determine the migration capacity of HUVEC cells after incubation with different CMs, we performed wound healing assays. HUVEC cells were grown, wounded, and treated with hRAN-, hRAT-, VEGF control- (positive control), and M199 control- (negative control) CMs. After 6 and 12 h of incubation, hRAT-CMs significantly enhanced HUVEC migration vs. hRAN-CMs and M199 control-CMs (*p* < 0.05 and *p* < 0.0001 respectively, Figures 3a,b). However, the positive control enhanced HUVEC migration, vs. hRAN-CMs and M199 control CMs, only after 12 h (*p*-value < 0.001).

### 3.4 hRAT-CMs promoted transmigration of ECs

**Figure 4:**
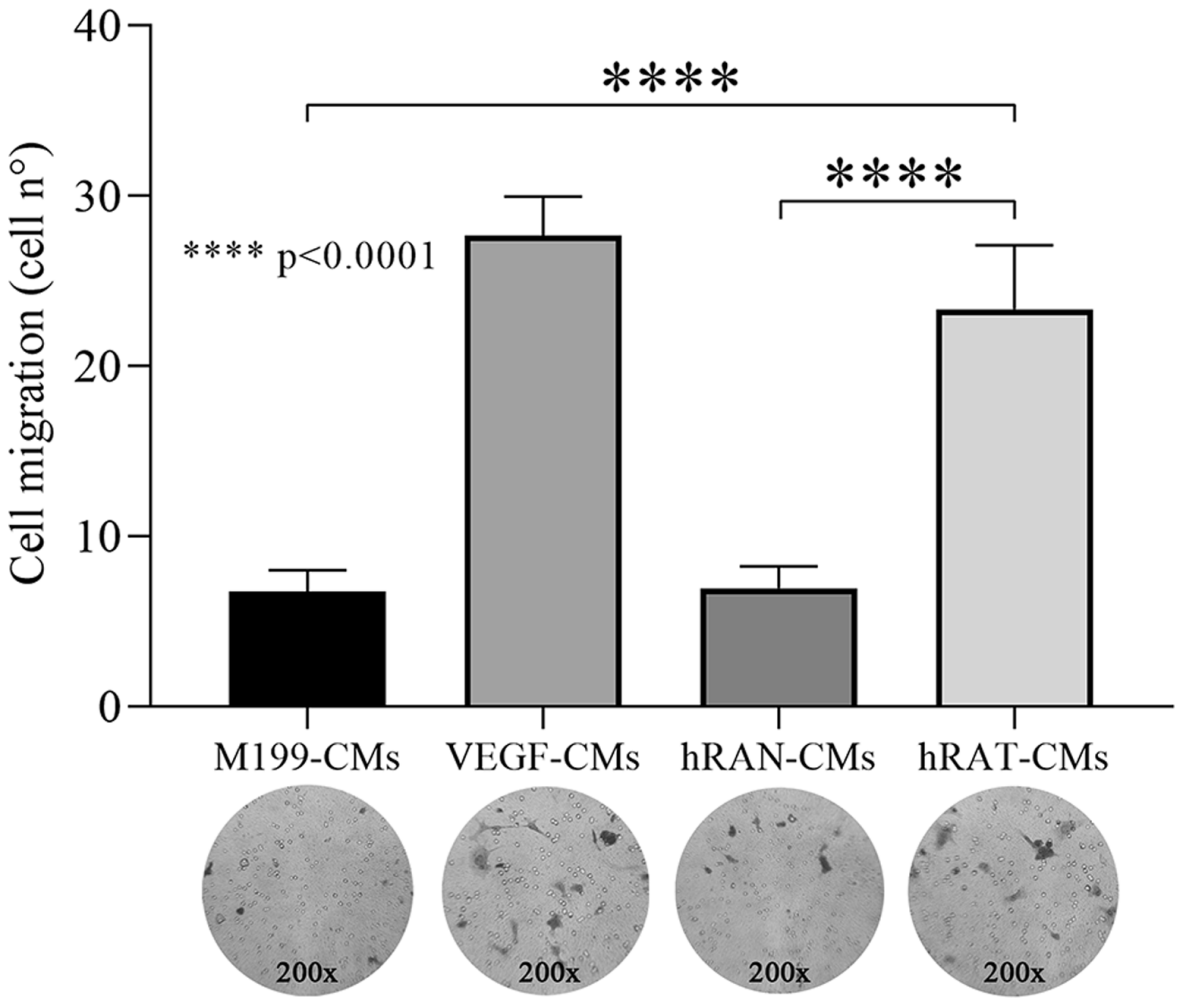
Effect of CMs from hRAN and hRAT on migration of HUVEC cell by transwell assay. Transmigration of HUVEC cells treated with hRAN-, hRAT-, VEGF-, or control-CMs through a porous membrane. Horizontal bars represent the geometric mean of each data set and vertical bars indicate SEM. ****p < 0.0001 hRAT-CMs vs. hRAN-CMs and M199-CMs. The *n* corresponds to six patient samples from each group, and the experiments were performed in triplicate.

To evaluate the transmigration capacity of HUVEC cells, due to chemoattractant factors secreted by AT, we performed a Transwell assay. HUVEC cells were grown with complete growth medium and treated with hRAN-, hRAT-, VEGF- (positive control) and M199- (negative control) CMs. After 18 h of incubation, hRAT-CMs significantly enhanced HUVEC transmigration, through the insert, vs. hRAN- and M199-CMs (*p* < 0.0001, Figure 4).

### 3.5 hRAT-CMs significantly increased tube formation by HUVEC cells compared to hRAN-CMs

**Figure 5:**
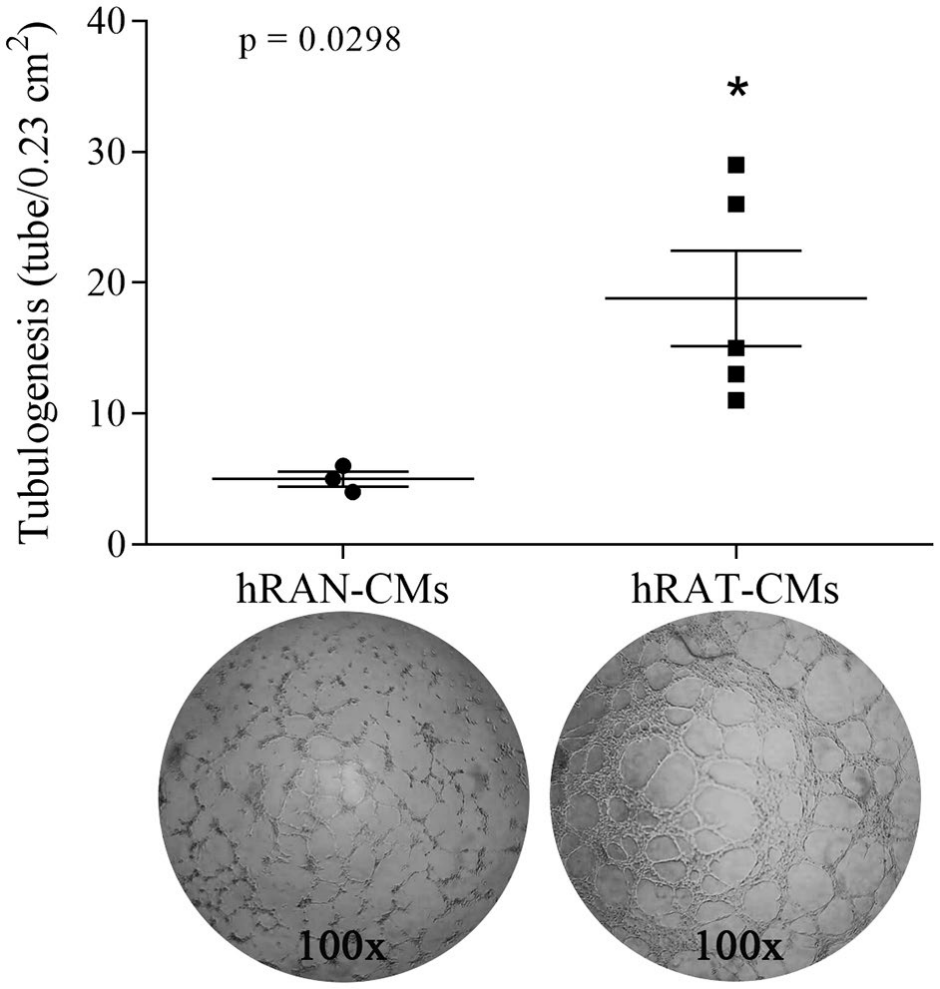
Tubulogenesis assays. Three-dimensional vessel formation of HUVEC cells in Geltrex® coated *μ*-slides incubated with hRAN-CMs (*n* = 3) and hRAT-CMs (*n* = 5). Quantification of tubular structures was performed by counting the number of tubes/0.23 cm^2^. Horizontal bars represent the geometric mean of each data set and vertical bars indicate SEM. *p < 0.05.

To evaluate the angiogenic activity of hRAT- and hRAN-CMs, we performed a tubulogenesis assay to model the formation of three-dimensional vessels. A VEGF-CM group for tubulogenesis was performed as a positive control of the assay, in order to evaluate whether HUVEC cells were indeed able to form new tubuli. This group showed a very significant increase of tubulogenesis compared to the hRAN-CM (*p* < 0.0001, data not shown). In addition, we observed that hRAT-CMs significantly increased the formation of ECs tubular networks compared to hRAN-CMs (Figure 5, *p* < 0.05).

## 4 Discussion

Clear cell renal cell carcinoma (ccRCC) is the most common form of renal cell carcinoma and represents one of the most lethal urological malignancies (28). Indeed, distant metastasis is the major cause of ccRCC-associated mortality. The molecular mechanisms involved in ccRCC metastasis remain to be fully understood (29, 30). One of the earliest oncogenic events in the majority of ccRCC is the loss of function of tumor suppresor gene VHL. This inactivation leads to constitutive stabilization of HIF-1α and HIF-2α, promoting activation of several oxygen-independent hypoxic transcriptional programs and, consequently, inducing angiogenesis, epithelial-to-mesenchymal transition, invasion, and metastatic spread (31). VEGF is a potent stimulator of endothelial cell proliferation and angiogenesis and is expressed by adipocytes and adipose stem cells (ASCs) (32). HIF1α and HIF2α are expressed in different tissues and regulate target genes involved in angiogenesis, cell proliferation and inflammation, and their expression is associated with different disease stages. HIFs have been widely studied respect to their involvement in cancer, and HIF2α-specific inhibitors are being investigated in clinical trials for the treatment of kidney cancer, among other cancer types (24).

Recently, our group demonstrated that conditioned media (CM) from human renal adipose tissue from patients with renal tumors (hRAT) increased the migration of tumor and non-tumor renal epithelial cells compared to CM from normal adipose tissue (hRAN) (14). Next, we showed that hRAT-CMs increase the expression of mesenchymal markers, like desmin, N-cadherine and vimentin, in both tumor and non-tumor renal epithelial cells and that AT can stimulate the epithelial-mesenchymal transition (EMT) and therefore the metastatic capacity of cells (15). Thus, in this work we evaluated whether human renal peritumoral adipose tissue is capable of stimulating angiogenesis and migration in the tumor microenvironment, thus favoring the growth and metastatic capacity of renal tumor cells.

Adipose tissue is rich in vasculature and secretes, in addition to adipocytokines such as leptin (14), interleukins, growth factors like fibroblats growth factor (FGF) and proangiogenic factors such as angiopoietin 2 and VEGF (33, 34). We show that hRAT express higher amounts of HIF-1α and HIF-2α mRNA compared to hRAN (Figures 1a,b). HIF-1α can induce VEGF overexpression and promote cancer progression (35). Although we did not find significant differences in gene expression of VEGF mRNA between hRAT and hRAN (Figure 1c), we demonstrated, by ELISA, that hRAT secretes greater amounts of VEGF protein than hRAN (Figure 2, *p* = 0.052), similar results were found by Sarkanen et al. (33) in adipose tissue extracts.

We then set out to assess whether this differential expression of angiogenic factors, by the different types of AT, was correlated with a different response from endothelial cells and their ability to form new blood vessels. We observed that hRAT-CMs increased both the migration of HUVECs by wound healing and transwell assays (Figures 3, 4, respectively). Surprisingly, we observed that HUVECs showed significant migration, measured as a smaller wound area, after 6 h of incubation with hRAT-CMs; while the closure was significant only after 12 h of incubation with the VEGF-CMs control (positive control). This may be due to the fact that hRAT-CMs not only contain higher levels of VEGF (Figure 2), but may also harbor other, yet unidentified, soluble factors that contribute to endothelial cell migration. We have previously shown that peritumoral adipose tissue expresses and secretes greater amounts of leptin than non-tumorous perirenal adipose tissue. Leptin has a proangiogenic role by stimulating the JAK/STAT3 pathway in vascular endothelial cells and by stimulating the production of VEGF (36). In addition to VEGF, other factors present in hRAT-CMs could be responsible for the observed pro-angiogenic effects. For example, Wagner et al. (37, 38) demonstrated the importance of macrophages present in peritumoral adipose tissue and their key role in the secretion of IL-6, postulating this interleukin as a possible angiogenic factor and inducer of endothelial cell proliferation. It would be interesting to evaluate the presence of macrophages in perirenal adipose tissue fragments from healthy patients and patients with renal cancer, as well as the expression of IL-6 in CMs.

Regarding the tubulogenic capacity of CMs we found that hRAT-CMs significantly increased tube formation by HUVEC cells compared to hRAN-CMs. Our results correlate with Saiki et al. (39), Rubina et al. (40), and Verseijden et al. (41) who have reported that adipose stem cells (ASCs) and 3 T3-L1 cell lines (all adipocyte cell lineage) promote tube formation by HUVEC cells.

The aforementioned authors have shown proangiogenic effects of adipocyte cell cultures, but there is a lack of information about the proangiogenic capacity of mature adipose tissue in a cancer context. This is one of the first studies to demonstrate that conditioned media from peritumoral renal adipose tissue can stimulate endothelial cell migration and tubulogenesis *in vitro*, suggesting the presence of soluble factors—such as VEGF—that may contribute to angiogenesis and, potentially, tumor progression. It has been shown that lymphangiogenesis may contribute to tumor progression by acting as a pathway for metastatic spread (42), so we believe it is necessary to conduct further studies in this regard.

## 5 Conclusion

We show that conditioned media derived from peritumoral renal adipose tissue contains higher levels of VEGF and promotes angiogenic responses in HUVEC cell lines. These findings suggest that soluble factors secreted by peritumoral adipose tissue may contribute to angiogenesis within the tumor microenvironment and potentially influence renal tumor progression.

## Data Availability

The original contributions presented in the study are included in the article/supplementary material, further inquiries can be directed to the corresponding author.
